# Development of a Health-Related Quality of Life Tool for Adolescents and Young Adults With Cancer

**DOI:** 10.1001/jamanetworkopen.2025.49071

**Published:** 2025-12-19

**Authors:** Samantha Claire Sodergren, Olga Husson, Silvie Janssen, Gudrun Elin Rohde, M. Jamal Hossain, Haneen Abaza, Ali Alkan, Amal Al-Omari, Irit Ben-Aharon, Line Bentsen, Marianne G. Guren, Georgios Ioannidis, Hiroto Ishiki, Michael Koehler, Emma Lidington, Iwona Ługowska, Fiona McDonald, Manjunath Nookala Krishnamurthy, Chana Korenblum, Alessandra Majorana, Nikolaos Memos, Maria Otth, Helle Pappot, Marta Pérez-Campdepadrós, Duska Petranovic, Diana Richter, Jelena Roganovic, Katrin Scheinemann, Anna Sikora-Koperska, Dan Stark, Niki Stavrou, Serdar Turhal, Jeanette Winterling, Noam Yarom, Anne-Sophie Emma Darlington

**Affiliations:** 1 School of Health Sciences, University of Southampton, Southampton, United Kingdom; 2 Department of Medical Oncology, Netherlands Cancer Institute – Antoni van Leeuwenhoek Hospital, Amsterdam, Netherlands; 3 Department of Surgical Oncology, Erasmus MC Cancer Institute, Erasmus University Medical Center, Rotterdam, Netherlands; 4 Department of Public Health, Erasmus MC Cancer Institute, Erasmus University Medical Center, Rotterdam, Netherlands; 5 Department of Medical Oncology, Erasmus MC Cancer Institute, Erasmus University Medical Center, Rotterdam, Netherlands; 6 Department of Health and Nursing, University of Agder, Agder, Norway; 7 Department of Clinical Research, Sorlandet Hospital, Kristiansand, Norway; 8 Marie Curie Palliative Care Research Department, Division of Psychiatry, University College London, London, United Kingdom; 9 Scientific Affairs and Research, King Hussein Cancer Center, Amman, Jordan; 10 Department of Medical Oncology, Muğla Sıtkı Koçman University, Muğla, Turkey; 11 Rambam Health Care Campus, Fishman Oncology Center, Haifa, Israel; 12 Department of Clinical Medicine, Copenhagen University Hospital - Rigshospitalet, Copenhagen, Denmark; 13 Department of Oncology, Oslo University Hospital, Oslo, Norway; 14 Institute of Clinical Medicine, University of Oslo, Oslo, Norway; 15 Oncology Department, Nicosia General Hospital, State Health Services Organization, Nicosia, Cyprus; 16 Department of Palliative Medicine, National Cancer Center Hospital, Tokyo, Japan; 17 Specialty Practice for Psycho-oncology, Magdeburg, Germany; 18 Centre for Cancer Screening, Prevention, and Early Diagnosis, Queen Mary University of London, London, United Kingdom; 19 Department of Soft Tissue/Bone Sarcoma and Melanoma, Maria Sklodowska-Curie National Institute and Oncology Centre, Warsaw, Poland; 20 Canteen, Sydney, New South Wales, Australia; 21 University of Sydney, Sydney, New South Wales, Australia; 22 Advanced Centre for Treatment, Research and Education in Cancer, Kharghar, Maharashtra, India; 23 HomiBhabha National Institute, Mumbai, India; 24 Division of Adolescent Medicine, Hospital for Sick Children and Princess Margaret Cancer Centre, Toronto, Ontario, Canada; 25 Department of Medical and Surgical Specialties, Radiological Sciences and Public Health, University of Brescia, Brescia, Italy; 26 National and Kapodistrian University of Athens, Hippocratio General Hospital, Athens, Greece; 27 Division of Oncology-Hematology, Children’s Hospital of Eastern Switzerland St. Gallen, Switzerland; 28 Department of Oncology, University Children’s Hospital Zurich, Zurich, Switzerland; 29 Pediatric Cancer Centre, Hospital Sant Joan de Déu, Barcelona, Spain; 30 Department of Child and Adolescent Mental Health, Hospital Sant Joan de Déu, Barcelona, Spain; 31 Clinical Hospital Center Rijeka, Medical Faculty University of Rijeka, Rijeka, Croatia; 32 Department of Medical Psychology and Medical Sociology, University Medical Center Leipzig, Leipzig, Germany; 33 Department of Pediatric Hematology and Oncology, Children’s Hospital Zagreb, Zagreb, Croatia; 34 Faculty of Health Sciences and Medicine, University of Lucerne, Lucerne, Switzerland; 35 School of Medicine, University of Leeds, Leeds, United Kingdom; 36 Department of Medical Oncology, Anadolu Medical Center, Istanbul, Turkey; 37 Center of Hematology, Karolinska University Hospital, Stockholm, Sweden; 38 Unit of Oral Medicine, Sheba Medical Center, tel-Hashomer, Israel; 39 Tel-Aviv University, Tel-Aviv, Israel

## Abstract

**Importance:**

A diagnosis of cancer during adolescence and young adulthood disrupts key developmental stages, affecting multiple domains of health-related quality of life (HRQOL). To provide a comprehensive assessment of the HRQOL issues of adolescents and young adults (AYAs) with cancer, a questionnaire dedicated to this age group is needed.

**Objective:**

To develop an HRQOL questionnaire, as a supplement to the European Organisation for Research and Treatment of Cancer (EORTC) QLQ-C30, that measures issues of relevance and importance to AYAs with cancer.

**Design, Setting, and Participants:**

This survey study was informed by a systematic review of AYA oncology literature published through December 31, 2015, and interviews with AYAs with cancer and health care professionals (HCPs) between February 2015 and February 2024. Participants were recruited from 19 cancer treatment centers in Europe, Asia, and various countries. Participants included HCPs providing care to AYAs with cancer and AYAs aged 14 to 39 years who were receiving treatment for cancer, had completed treatment within the past 12 months, or were receiving palliative care. Data analysis for the survey responses was performed from May to November 2024.

**Main Outcomes and Measures:**

The primary study outcome was the development of an HRQOL questionnaire that assesses issues of importance and relevance to AYAs with cancer. A draft questionnaire was created and tested for relevance and importance. Exploratory factor analysis identified questionnaire subscales. Internal consistency of items within HRQOL domains was checked using Cronbach α.

**Results:**

A total of 253 AYAs (mean [SD] age, 25.5 [7.5] years; 129 males [51%]) completed the draft questionnaire, and 28 HCPs from 20 countries were also involved in this survey study. Pilot testing of the draft questionnaire resulted in a 30-item questionnaire covering 5 subscales (activity limitations and life disruptions, worry about cancer and the future, self-esteem, relationships, and positive changes) and 9 single questions. Reliability testing indicated good internal consistency, with Cronbach α ranging from 0.659 (relationships subscale) to 0.770 (positive changes subscale). Questions about distinguishing between important and nonimportant things and motivation to live life to the full were the most relevant to 89% (211 of 252) and 84% (224 of 252) of AYAs, respectively. Concerns about appearance changes and fertility were identified as priority questions by 24% (21 of 86) of AYAs.

**Conclusions and Relevance:**

In this survey study, a measurement tool was developed and tested that captures HRQOL issues of importance and relevance to AYAs from diverse cultures and with different cancer and treatment types. This HRQOL questionnaire could be a reliable and valid tool for both clinical trials and practice.

## Key Points

**Question:**

Can a cross-cultural questionnaire be developed to comprehensively assess health-related quality of life (HRQOL) in adolescents and young adults (AYAs) with cancer?

**Findings:**

In this survey study involving 253 AYAs from 19 countries, a 30-item HRQOL questionnaire was developed that assesses HRQOL issues of importance and relevance to AYAs from diverse cultures with different cancer and treatment types.

**Meaning:**

Findings of this study suggest that the HRQOL questionnaire provides a reliable, valid tool for use in both clinical trials and practice.

## Teaser

This survey study describes the process of creating and testing an instrument for assessing quality-of-life concerns of patients with cancer aged 14 to 39 years with input from these patients and their clinicians.

## Introduction

Adolescents and young adults (AYAs) aged 15 to 39 years^1,2^ are at a critical developmental stage characterized by substantial physical, cognitive, and psychosocial changes as they transition to adulthood. AYAs navigate multiple challenges as they forge career pathways, establish autonomy from family, and explore intimacy and sexuality.^3^ Cancer complicates the negotiation of these tasks. AYAs have worse outcomes compared with children and older adults due to aggressive disease biological process, diagnostic delays given low suspicion of cancer, and poorer clinical trial enrollment.^1,4,5^ Their care needs are often unmet due to a lack of specialized AYA health care practitioners and units.^6^ For AYAs with cancer, the implications of cancer and its treatment for multiple domains of life—known as health-related quality of life (HRQOL)—is likely to be distinct and more substantial than for their younger and older counterparts.^1^

Patient-reported outcome (PRO) assessments provide detailed insight into the experience of cancer and its treatment allowing for the monitoring and management of adverse effects, contributing to decision-making and informing supportive care interventions. HRQOL is a core outcome in clinical trials, although PRO end points in trials for AYAs are often absent, due in part to a lack of appropriate measurement tools for AYAs and no consensus regarding the standardized assessment of HRQOL for these patients.^7^

Existing studies mostly use general cancer measures such as the European Organisation for Research and Treatment (EORTC) core measure: the 30-item EORTC QLQ-C30,^8^ which is not validated for AYAs. Adapted tools, such as the teen and young adult versions of the Pediatric Quality of Life Inventory,^9^ rarely involve AYAs in development.^10^ Recent efforts (eg, the Patient-Reported Outcomes Measurement Information System [PROMIS]^11^ and the Patient-Reported Outcome–Common Terminology Criteria for Adverse Events^12^) partly address this lack of involvement, but AYA-specific measures remain limited.^13^

An urgent need for the development of robust HRQOL measures covering the AYA age spectrum was initially highlighted as part of the landmark report by an AYA oncology review group^1,14^ and has been subsequently reiterated.^7,13,15,16^The EORTC Quality of Life Group (QLG) has an international reputation for the development of cancer HRQOL measurement tools. The QLQ-C30^8^ was designed to assess multiple domains of HRQOL (physical, social, and emotional) for all patients with cancer regardless of tumor site, treatment modality, or age group. The EORTC QLG advocates a modular approach to the development of questionnaires to supplement the QLQ-C30, with questions tailored to specific HRQOL concerns of the target population.

The aim of our research is to develop an HRQOL questionnaire, as a supplement to the QLQ-C30,^8^ that measures issues of relevance and importance to AYAs with cancer. For the purposes of this work, we operationalized the age range for AYAs as 14 to 39 years, in line with clinical service configuration at the onset of the project.

## Methods

Following the EORTC QLG questionnaire development guidelines,^17^ we conducted this mixed-methods, cross-cultural survey study across 3 phases. We followed the American Association for Public Opinion Research (AAPOR) reporting guideline.^18^ Ethical and research governance approvals were obtained at the lead academic treatment center (University of Southampton Research Ethics Committee^15^) and each collaborating center across 20 countries. All participants provided written informed consent. We adhered to ethical guidelines to ensure confidentiality, voluntary participation, and the right to withdraw.

Participants included AYAs aged 14 to 39 years at the time of study enrollment who were currently receiving treatment for cancer, had completed cancer treatment within the past 12 months, or were receiving supportive care for incurable cancer. AYAs who had completed treatment more than 12 months earlier and were cancer-free were excluded. Health care professionals (HCPs) involved in the care of AYAs with cancer were also invited to participate.

Purposive sampling was conducted to ensure good representation of AYAs according to age, sex, cancer diagnosis, and treatment type and intent (palliative and curative). Furthermore, participants were recruited from multiple cancer centers covering different geographical regions in Europe, Asia, and various countries to further strengthen sampling diversity in terms of languages, cultures, and health care systems. The sampling matrices for each phase of the work are outlined in the study protocols and are available on request.

### Phase 1: HRQOL Issue Generation and Issue Review

In phase 1, information collected from a systematic review of AYA oncology literature published through December 31, 2015, as reported elsewhere,^13^ and concepts elicited from AYAs and HCPs were used to create an exhaustive list of HRQOL issues. Interviews involved AYAs from 10 countries and were conducted between February 2015 and November 2019. The target sample size was informed by the EORTC QLG guidelines^17^ and set at 65 participants.

Interviews followed a semistructured schedule (eAppendix 1 in Supplement 1) and asked AYAs to discuss how their lives had changed since their diagnosis, followed by a consideration of issues extracted from the literature review^13^ and the QLQ-C30. A case report form covering self-reported sociodemographic (including ethnicity) and clinical characteristics extracted from medical notes was completed to help describe the sample and determine its representativeness. HCPs from 6 countries were also asked to comment on the HRQOL issues experienced by AYAs they treated. Interview summaries were analyzed using the principles of thematic analysis and involving a team of 5 reviewers, and coding assumptions were continually reviewed.^19^

A separate group of AYAs from 6 countries and HCPs from 3 countries rated the generated and QLQ-C30 issues for relevance (yes or no) and importance or bothersome quality (1 [not at all], 2 [a little], 3 [quite a bit], or 4 [very much]). The group then nominated the top-10 priority issues and issues to be removed (omissions).

### Phase 2: Drafting the Questionnaire

Issues were refined for redundancy, relevance, and applicability to clinical practice or trials and then matched to items in the EORTC Item Library^20^ or formulated as new questions. The provisional questionnaire was reviewed by collaborators and an AYA (aged 16-32 years) advisory panel.

### Phase 3: Pilot Testing the Questionnaire With AYAs Aged 14 to 39 Years

The questionnaire was translated into 17 languages and pilot tested with AYAs from 19 countries, representing diverse geographical regions, languages, and cultures. Target sample size (200-250) was informed by the EORTC QLG guidelines.^17^ Participants completed the QLQ-C30 and the draft questionnaire and offered feedback on question wording, acceptability, relevance, importance, and omissions (eAppendix 2 in Supplement 1). Background sociodemographics and clinical data were collected for all participants.

### Decision Rules

The following decision rules of the EORTC QLG guidelines^17^ were applied: (1) mean rating score higher than 1.5; (2) range higher than 2 points; (3) prevalence ratio greater than 30%; (4) no floor or ceiling effects; (5) compliance, defined as at least 95% response to the item; (6) more than 60% of rating as relevant or important; (7) mean importance score higher than 1.5; and (8) no significant concerns expressed. Items meeting at least 5 of the 8 criteria were considered for retention.

### Statistical Analysis

Descriptive statistics were used to identify response distributions, item insensitivity, and missing responses. Mean and range of item scores were calculated as well as prevalence ratios (the number of participants who scored an item as 2 [a little], 3 [quite a bit], or 4 [very much] divided by the total number of respondents who completed that item, multiplied by 100). For conditional questions, mean and prevalence ratio scores were calculated according to the number of AYAs responding yes. Percentages of participants indicating that an item was relevant or at least “a little” important were determined.

To identify overlapping questions, Spearman correlations (*r*) were performed, with correlation coefficients of 0.70 or greater indicative of items measuring similar constructs. Exploratory factor analysis was conducted using Promax rotation and maximum likelihood estimation to identify the questionnaire’s underlying factor structure. The suitability of the data for factor analysis was confirmed using the Kaiser-Meyer-Olkin measure of sampling adequacy (threshold of 0.5) and Bartlett test of sphericity for significance. The scree plot and parallel analysis were used to determine the optimal number of factors to retain. Factor loadings of 0.3 or higher were considered significant. Internal consistency of identified factors (subscales) was assessed using Cronbach α, with values of 0.7 or higher considered indicative of acceptable reliability. Spearman correlations were conducted for AYA questionnaire and QLQ-C30 scores to identify redundancy and convergent validity. Data analysis for the survey responses was performed from May to November 2024 using Stata, version 14.1 (StataCorp LLC).

## Results

### Phase 1

Interviews included 45 AYAs (24 males [53%]; mean [SD] age, 20.3 [2.8] years). Leukemia (12 [28%]) and lymphoma (8 [18%]) were the most common of the 12 cancer types presented, with time since diagnosis between 1 month and 7 years. Most participants (35 [78%]) were receiving curative intent treatment. The sociodemographic and clinical characteristics of the sample are published elsewhere^21^ and reflect the epidemiologic distribution of cancer types in the population as well as good distribution across countries with different language groups.

Eighty-one issues covering 12 categories were captured: symptoms (pain, nausea, and vomiting), from 38 AYAs (84%); activity restrictions (education and hobbies), from 39 AYAs (87%); disrupted life plans, from 13 AYAs (29%); social functioning (loss of friends), from 41 AYAs (91%); emotional functioning (depression and anxiety), from 29 AYAs (64%); body image, from 16 AYAs (36%); self-appraisals (greater maturity and bravery), from 21 AYAs (47%); outlook on life (altered priorities and increased motivation to achieve), from 15 AYAs (33%); lifestyle (diet and avoidance of infections), from 8 AYAs (18%); treatment-related (age-appropriate information and treatment burden), from 14 AYAs (31%); fertility, from 11 AYAs (24%); and financial concerns, from 9 AYAs (13%). These issues were combined with those captured from the literature review^13^ and then reviewed for content overlap (also considering QLQ-C30 questions), leaving 77 issues for review.

An additional group of 29 young adults (19 females [66%]; mean [SD] age, 31.6 [4.4] years) (eTable 1 in Supplement 1) added 10 issues to the list (eTable 2 in Supplement 1). Fifty-eight of the 77 issues (75%) presented were discussed spontaneously by these participants. The physical impact of cancer and its treatment was mentioned by 18 young adults (62%). All 20 HCPs interviewed (eTable 3 in Supplement 1) judged the issues as relevant and identified no omissions.

A total of 33 AYAs and 8 HCPs rated the issues and confirmed their relevance. The characteristics of AYAs and their ratings have been reported elsewhere^22^; eTable 3 in Supplement 1 presents HCP characteristics.

### Phase 2 

Decisions relating to the 87 issues are provided in eTable 2 in Supplement 1. Of these issues, 41 were removed due to overlap, redundancy, or inapplicability, resulting in 50 questionnaire items (eAppendix 2 in Supplement 1). Of these questions, 10 were selected from the EORTC Item Library,^20^ 14 were adapted, and 26 were new. Items included 12 positive changes and 8 conditional questions.

### Phase 3 

#### Participants

The draft questionnaire was completed by 253 AYAs, of whom 216 (85%) completed relevance and importance ratings. These participants had a mean (SD) age of 25.5 (7.5) years and included 129 males (51%), with 54 (21%) reporting a sarcoma diagnosis. Mean (SD) time since diagnosis was 17.7 (8.0) months, and most AYAs were currently receiving treatment (205 [81%]) and many (199 [79%]) received treatment with curative intent. Table 1 presents the sociodemographic and clinical characteristics of the sample, revealing the diversity in representation.

**Table 1.** Sociodemographic and Clinical Characteristics of Adolescents and Young Adults Who Pilot Tested the Draft Questionnaire

Characteristic	AYAs, No. (%) (N = 253)
Country	
Australia	4 (2)
Croatia	16 (6)
Cyprus	7 (3)
Denmark	25 (10)
Germany	7 (3)
Greece	4 (2)
Israel	22 (9)
India	10 (4)
Italy	3 (1)
Japan	19 (8)
Jordan	25 (10)
Netherlands	12 (5)
Norway	8 (3)
Poland	10 (4)
Spain	9 (4)
Sweden	6 (2)
Switzerland	6 (2)
Turkey	27 (11)
United Kingdom	33 (13)
Sex	
Female	124 (49)
Male	129 (51)
Age group, y	
14-18	56 (22)
19-25	98 (39)
26-39	99 (39)
Ethnicity^a^	
Arab	25 (10)
Asian	33 (13)
White	167 (66)
Multiracial	6 (2)
Other^b^	18 (7)
Not specified	3 (1)
Educational level	
<Compulsory education	24 (10)
Currently attending full-time compulsory education	36 (14)
Compulsory education	67 (27)
Postcompulsory education: college, vocational school	46 (18)
University	68 (27)
Not specified	12 (5)
Employment status	
Full-time	59 (23)
Part-time	29 (12)
Homemaker	14 (6)
Sick leave	53 (21)
Disability	4 (2)
None	74 (29)
Other^c^	16 (6)
Living situation	15 (6)
Living alone	23 (9)
Living with parents	144 (57)
Living with a partner	66 (26)
Living with a partner at parents’ house	3 (1)
Living with others (friends, relatives)	16 (6)
Not specified	1 (0)
Cancer diagnosis	
Biliary tract	1 (0)
Bladder	1 (0)
Brain and CNS	12 (5)
Breast	25 (10)
Bone	2 (1)
Colorectal	9 (4)
Gastric	1 (0)
Gynecological	11 (4)
Head and neck	13 (5)
Leukemia	31 (12)
Lymphoma	49 (19)
Melanoma	6 (2)
Multiple myeloma	1 (0)
Neuroendocrine	2 (1)
Pancreatic	4 (2)
Sarcoma	54 (21)
Testicular (germ cell)	27 (11)
Thyroid	3 (1)
Disease stage	
Localized	116 (46)
Locally advanced	1 (0)
Metastatic	81 (32)
Not applicable (eg, leukemia)	51 (20)
Not specified or unknown	4 (2)
Treatment status	
Currently on or about to start treatment	205 (81)
Completed treatment in the past 12 mo	33 (13)
Supportive or palliative care	11 (4)
Treatment scheduled but not yet started	4 (2)
Treatment intent	
Curative	199 (79)
Palliative	50 (20)
Not specified	4 (2)
Treatment type	
Chemotherapy	188 (74)
Hormonal therapy	15 (6)
Immunotherapy	23 (9)
Radiotherapy	29 (12)
Stem cell treatment	7 (3)
Surgery	126 (50)
Targeted therapy	25 (10)
Other^b^	4 (2)
Comorbidity	
Yes	64 (25)
Type	
Bladder	3 (1)
Blood disorder	3 (1)
Diabetes	2 (1)
Depression or anxiety	12 (5)
Eye	2 (1)
Kidney	7 (3)
Genetic	3 (1)
Heart	4 (2)
Hypertension	2 (1)
Inflammatory bowel disease	2 (1)
Respiratory	8 (3)
Rheumatic	2 (1)
Skin	6 (2)
Thyroid	6 (2)
Other: not specified	9 (4)
ECOG performance status	
0: Fully active	126 (50)
1: Restricted in physically strenuous activity	80 (32)
2: Ambulatory and capable of all self-care but not work activities	31 (12)
3: Capable of only limited self-care	9 (4)
4: Completely disabled	3 (1)
Not specified	4 (2)

Abbreviations: AYA, adolescent and young adult; CNS, central nervous system; ECOG, Eastern Cooperative Oncology Group.

^a^ Ethnicity were self-reported and obtained from medical records.

^b^ Other category was not specified.

^c^ Other employment status included furlough and student.

#### Performance of Questions

All questions were completed by at least 250 AYAs (99%) (Table 2), and there was no evidence of floor or ceiling effects. Only 1 question on loss of friendships did not meet the threshold for prevalence (27% [68 of 252] rated this item at least a 2 [a little bit]). Questions about distinguishing between important and nonimportant things and enhanced motivation to live life to the full were relevant to 89% (211 of 252) and 84% (224 of 252) of AYAs, respectively. Concerns about appearance changes and ability to have children were priority questions for 24% (21 of 86) of AYAs.

**Table 2.** Item Performance Following Phase 3 Pilot Testing of the Draft Questionnaire

Question item	No. of valid entries	Mean (SD) score^a^	Prevalence ratio of scores^b^	Relevance score, %^c^	Mean (SD) importance^d^	Importance prevalence, %^e^	Priority, No. (%)^f^	No. of participants who regarded the question as upsetting or inappropriate	No. of participants who regarded question as confusing or difficult to understand
1. Have you lacked energy?	252	2.45 (0.95)	84	93	3.07 (0.85)	71	13 (15)	0	1
2. Have you been watching yourself closely for any new symptoms of disease?	253	2.38 (1.09)	73	80	2.99 (0.96)	72	8 (9)	0	0
3. Have you had mobility problems?	250	1.85 (1.00)	52	75	2.79 (0.97)	63	2 (2)	0	2
4. Have you had problems taking part in social activities?	252	2.18 (1.10)	64	77	2.98 (0.94)	68	11 (13)	0	1
5. Have you felt isolated from your friends?	253	1.95 (1.05)	55	65	2.95 (1.00)	65	8 (9)	1	1
6. Have you worried about people treating you differently?	252	1.87 (0.99)	52	66	2.69 (0.99)	55^g^	3 (3)	0	2
7. Have you felt bored?	251	2.24 (1.12)	65	72	2.76 (0.85)	56^g^	6 (7)	0	3
8. Have you felt stressed?	253	2.27 (1.02)	74	80	2.95 (0.86)	66	11 (13)	0	0
9. Have you spent time thinking about your disease?	253	2.46 (1.03)	80	86	2.97 (0.91)	66	8 (9)	1	3
10. Have you worried about your health in the future?	252	2.56 (1.04)	81	88	3.07 (0.91)	72	20 (23)	4	0
11. Have you worried about dying?	250	1.84 (0.99)	51	66	2.90 (1.00)	63	6 (7)	19	0
12. Have you worried about getting infections?	251	2.12 (1.10)	62	73	2.80 (0.98)	62	4 (5)	1	1
13. Have you felt restricted in the types of food and drink you can have?	252	2.04 (1.12)	56	73	2.81 (0.97)	60	8 (9)	0	1
14. Has your family been negatively affected?	251	2.09 (1.02)	64	76	3.02 (0.88)	70	13 (15)	3	10
15. Have your friends been negatively affected?	253	1.77 (0.90)	51	63	2.65 (0.91)	55^g^	10 (12)	2	8
16. Have you had to change your career plans?	253	2.14 (1.05)	64	75	2.86 (0.98)	62	14 (16)	0	3
17. Have you had problems making plans for the future?	252	2.36 (1.09)	72	76	2.91 (0.94)	65	14 (16)	0	3
18. Have you been dependent on others?	252	2.46 (1.11)	75	81	2.87 (0.94)	62	5 (6)	0	2
19. Have you lost friendships?	252	1.40 (0.75)^g^	27^g^	48^g^	2.52 (1.07)	49^g^	5 (6)	0	1
20. Has your romantic life been negatively affected?	244	1.74 (1.02)	43	51^g^	2.90 (1.06)	64	13 (15)	9	0
21. Has your sex life been negatively affected?	242	1.83 (1.11)	43	52^g^	2.88 (1.06)	64	15 (17)	12	1
22. Have you worried about changes to your appearance?	252	2.42 (1.09)	75	81	2.92 (0.97)	65	21 (24)	1	0
23. Have you lacked self-confidence?	251	1.93 (1.03)	55	71	2.65 (1.00)	55^g^	7 (8)	1	3
24. Have you felt that you have lost control over your life?	251	2.17 (1.06)	65	76	2.88 (0.99)	62	10 (12)	1	0
25. Have you become more negative about life?	252	1.76 (0.90)	50	65	2.68 (0.92)	56^g^	6 (7)	2	0
26. Have you worried about your disease or treatment causing you health problems in the future?	253	2.46 (1.03)	79	85	2.98 (0.88)	68	12 (14)	2	3
27. Have you been worried about your ability to have children?	252	2.12 (1.19)	55	65	2.99 (1.00)	69	21 (24)	4	1
28. Have you worried about passing cancer on to the next generation?	251	2.02 (1.04)	51	64	2.80 (1.06)	60	11 (13)	1	0
29. Have you felt that it is unfair that you became ill?	251	2.07 (1.20)	53	63	2.80 (1.08)	37	7 (8)	1	1
30. Has your relationship with any of your family members improved?	250	2.34 (1.11)	70	72	2.96 (0.92)	68	5 (6)	0	1
31. Has your relationship with any of your friends improved?	251	2.02 (1.04)	60	65	2.78 (0.95)	61	2 (2)	0	2
32. Have you made new friends?	252	1.73 (0.98)	43	55^g^	2.38 (0.95)	39^g^	4 (5)	0	3
33. Have you become more confident?	252	1.86 (1.03)	51	62	2.72 (0.92)	58^g^	3 (3)	0	4
34. Have you become mentally stronger?	251	2.40 (1.06)	75	80	2.86 (0.88)	65	5 (6)	0	3
35. Have you felt more mature?	251	2.29 (1.07)	70	73	2.72 (0.92)	57^g^	7 (8)	0	2
36. Have you made positive lifestyle changes (eg, healthy eating, exercise)?	251	2.22 (1.01)	70	78	2.78 (0.92)	61	6 (7)	0	1
37. Have you become more positive about life?	251	2.15 (1.06)	65	73	2.81 (0.93)	62	5 (6)	0	4
38. Have you felt more motivated to live life to the full?	252	2.63 (1.07)	80	84	2.97 (0.91)	70	7 (8)	2	4
39. Have you felt more motivated to achieve your personal goals?	252	2.40 (1.05)	75	77	2.89 (0.89)	66	7 (8)	0	3
40. Has your experience helped you to distinguish between important and non-important things in life?	252	2.90 (0.98)	89	89	3.15 (1.09)	77	17 (20)	0	5
41. Problem with weight gain	99	2.20 (1.03)	70	56^g^	2.64 (1.09)	56	5 (6)	1	0
42. Problem with having lost weight	144	2.15 (1.01)	69	69	2.51 (1.07)	46	8 (9)		1
43. Worry about ability to care for others	97	2.62 (0.98)	87	59^g^	2.81 (1.06)	58^g^	10 (12)	1	6
44. Problem with having to change where you live	61	2.56 (1.00)	85	36^g^	2.70 (1.10)	57^g^	2 (2)		0
45. Worry about impact on education	106	2.73 (1.03)	87	56^g^	2.73 (1.08)	54^g^	8 (9)	2	0
46. Worry about impact on job	139	2.74 (1.02)	86	65	2.94 (1.03)	70	15 (17)	3	0
47. Problem having to take regular medication	174	2.07 (1.04)	63	63	2.72 (1.07)	58^g^	3 (3)	0	1
48. Have your religious or spiritual beliefs weakened?	130	1.58 (1.52)	11^g^	57^g^	2.26 (1.10)	44^g^	1 (1)	2	0
49. Have your religious or spiritual beliefs strengthened?	130	2.37 (1.28)	60	75	2.82 (1.05)	64	3 (3)	2	0
50. Women only: problem with early menopause	50	2.72 (1.09)	84	63	3.16 (1.04)	73	4 (5)	0	1

^a^ Mean scores were calculated from total valid entries (for questions 41-50, score is conditional on the total number of people answering in the affirmative).

^b^ Prevalence ratio of scores was calculated from the number of respondents indicating 2 (a little), 3 (quite a bit), or 4 (very much) divided by the number of total respondents multiplied by 100.

^c^ Relevance scores were calculated from the number of participants completing relevance ratings. For questions 48 and 49, relevance was taken from the number of participants reporting that they had religious or spiritual beliefs.

^d^ Mean importance was calculated from the number of participants marking the question as relevant.

^e^ Importance prevalence was calculated from the number of respondents indicating 3 (quite a bit) or 4 (very much) divided by the number of respondents answering the question (ie, indicated as relevant).

^f^ Priority nominations were provided by 86 participants.

^g^ Did not meet the threshold.

Thirty-four of 253 AYAs (13%) identified at least 1 upsetting or inappropriate question, including worry about dying (19 of 250 [48%]). Questions about sexual and romantic relationships were regarded inappropriate by 12 (of 242 [29%]) and 9 (of 244 [22%]) participants, respectively. Clarification on question meaning was requested by 45 AYAs (18%).

Six of 253 AYAs (2%) recommended more questions on areas such as intimacy, caring for children, fertility, and emotional sequelae of cancer. Worry about telling others about cancer and treatment adverse effects, such as hair loss, were identified as missing.

#### Overlap With QLQ-C30

Lack of energy, mobility problems, and boredom correlated with QLQ-C30 questions from each subscale (eTable 5 in Supplement 2). Problems with social activities correlated with the QLQ-C30 questions about limitations in hobbies (Spearman *r* = 0.61) and interference with social activities (Spearman *r* = 0.64).

#### Redundancy Among Questions

Spearman correlations revealed that most questions were measuring distinct concepts (Spearman *r* < 0.50) (eTable 6 in Supplement 2). The highest correlation was between questions about family being negatively affected and about friends being negatively affected (Spearman *r* = 0.73). Questions on motivation to achieve personal goals and to live life to the full were also strongly correlated (Spearman *r* = 0.69).

#### Psychometric Analyses

The Kaiser-Meyer-Olkin score of 0.84 indicated suitable partial correlations, and the Bartlett test of sphericity confirmed the factorability of the correlation matrix (*P* < .001). Initially, eigenvalues greater than 1 suggested a 10-factor solution. However, a more parsimonious 6-factor solution was identified at the elbow point of the scree plot (Figure), accounting for 53% of the variance and providing a good conceptual fit. Table 3 displays the loadings for each question across the 6 factors: activity limitations (eg, mobility [loading, 0.84] and boredom [loading, 0.71]: factor 3); life disruptions (eg, plans for the future [loading, 0.78]: factor 6); worry about cancer and the future (eg, worry about future health [loading, 0.70] and dying [loading, 0.59]: factor 1); relationships (eg, with friends [loading, 0.76] and family [loading, 0.75]: factor 5); self-esteem (eg, lack of confidence [loading, 0.64] and changes to appearance [loading, 0.74]: factor 1); and positive changes (eg, priorities [loading, 0.45] and motivation to live life to the full [loading, 0.53]: factor 2).

**Figure.**
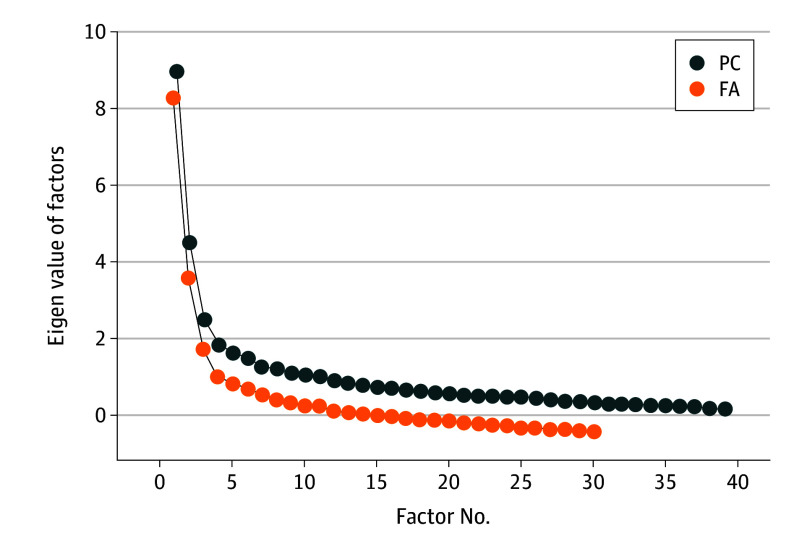
Exploratory Factor Analysis Scree Plot The elbow of the scree plot indicates a 6-factor solution. FA indicates factor analysis; PC, principal component.

**Table 3.** Factor Loadings From Exploratory Factor Analysis With Promax Rotation Allowing for Correlated Factors

Composite item	Factor 1	Factor 2	Factor 3	Factor 4	Factor 5	Factor 6
1. Lack of energy	0.06	0.06	0.53^a^	0.11	−0.06	−0.05
2. Watch closely for new symptoms	0.36^a^	−0.28	0.18	−0.01	0.19	0.00
3. Mobility problems	−0.12	−0.10	0.84^a^	−0.15	0.06	−0.06
4. Social activities	0.03	−0.02	0.62^a^	0.02	−0.02	0.07
5. Isolated from friends	−0.01	0.07	0.49^a^	0.13	−0.04	0.13
6. Worry people will treat you differently	0.28^b^	0.07^b^	0.28^b^	0.16^b^	−0.20^b^	−0.12^b^
7. Boredom	0.01	0.07	0.71	0.03	0.03	−0.21
8. Stress	0.43^a^	0.03	0.31	0.15	−0.06	−0.09
9. Long periods thinking about disease	0.73^a^	0.01	0.17	−0.01	0.11	−0.13
10. Worry about health in the future	0.70^a^	−0.02	0.13	0.02	0.22	0.04
11. Worry about dying	0.59^a^	−0.06	0.00	0.16	0.28	0.06
12. Worry about getting infections	0.13^b^	−0.16^b^	0.19^b^	0.24^b^	0.02^b^	0.08^b^
13. Restricted in food and drink	−0.10	0.01	0.35^a^	0.26	−0.12	0.15
14. Family negatively affected	−0.22	0.01	0.27	0.79^a^	0.01	0.01
15. Friends negatively affected	−0.05	0.04	−0.04	0.91^a^	0.02	0.09
16. Worry about changing career plans	0.14	0.03	−0.17	0.09	0.11	0.67^a^
17. Problems making plans for the future	0.13	0.01	0.07	−0.02	0.16	0.78^a^
18. Dependent on others	0.35^a^	−0.10	0.27	−0.20	0.01	0.13
19. Loss of friends	−0.02	−0.16	0.15	0.3	−0.13	−0.06
20. Romantic life negatively affected	0.32^b^	−0.02^b^	−0.01^b^	−0.05^b^	−0.34^a^^,^^b^	0.08^b^
21. Sex life negatively affected	0.41^a^	0.04	−0.09	0.00	−0.26	0.08
22. Worry about changes to appearance	0.74^a^	−0.04	0.03	−0.16	−0.14	−0.08
23. Lack of self-confidence	0.64^a^	0.15	−0.03	−0.03	−0.13	0.12
24. Loss of control over life	0.37^a^^,^^b^	0.13	0.05	0.09	−0.02	0.36^b^
25. More negative about life	0.47^a^	0.24	0.16	−0.01	−0.03	0.02
26. Worry about health problems caused by disease or treatment	0.76^a^	−0.10	−0.07	−0.07	0.09	0.23
27. Worry about ability to care for children	0.60^a^	−0.06	−0.15	−0.06	−0.05	0.04
28. Worry about passing cancer on to next generation	0.61^a^	−0.05	−0.19	0.03	0.03	0.00
29. Unfair	0.55^a^	0.16	0.01	−0.08	−0.11	−0.06
30. Improved relationships with family	0.00	0.22	−0.18	0.08	0.75^a^	0.23
31. Improved relationships with friends	0.03	0.13	0.08	−0.07	0.76^a^	0.08
32. New friends	0.18	0.42^a^	−0.04	−0.02	0.18	−0.19
33. More confident	0.21	0.70^a^	−0.14	0.09	0.00	0.00
34. Mentally stronger	−0.03	0.75^a^	−0.11	0.16	0.04	0.11
35. More mature	0.04	0.76^a^	−0.18	0.11	0.00	−0.07
36. Positive lifestyle changes	0.03	0.37^a^	0.04	0.00	0.06	−0.19
37. Positive about life	0.04	0.73^a^	0.10	−0.02	−0.03	0.09
38. Motivation to live life to the full	−0.18	0.60^a^	0.26	−0.18	0.04	0.08
39. Motivated to achieve personal goals	−0.11	0.53^a^	0.15	−0.29	0.05	0.22
40. Priorities	−0.19	0.45^a^	0.06	−0.01	0.18	0.05

^a^ Item has strong loading on the corresponding factor (factor loading >0.30), showing a meaningful association with that factor.

^b^ Item has weak loading (factor loading <0.30) or loads approximately equally on multiple factors, which may indicate the need for further evaluation or potential removal from the factor structure.

##### Revisions to the Questionnaire

No additional items were added to the questionnaire, with omissions identified as either covering existing items, non–AYA-specific items (ie, hair loss), or non–HRQOL-specific items (ie, satisfaction with care). Of the 50 questions tested, 25 were retained in their original format, 4 were reworded, and 2 were combined to create a reworded question. Fourteen were removed based on the decision rules, resulting in the 30-item EORTC QLQ-AYA30 (hereafter HRQOL questionnaire). Decisions made for each question are presented in eTable 4 in Supplement 1.

##### Final Questionnaire and Proposed Scale Structure

The subscales and composite items of the 30-item HRQOL questionnaire are presented in Table 4. Twenty-five questions use a 1-week time frame, 3 questions ask for reflection on the past 4 weeks, and 2 questions are general items. Internal reliability of each subscale was moderate to acceptable, with Cronbach α ranging from 0.659 (relationships subscale) to 0.770 (positive changes subscale) (Table 4). Although the exploratory factor analysis favored a 6-factor solution, questions measuring life disruption include a not-applicable response option and are treated as single items. The final instrument includes 5 subscales and 9 single items.

**Table 4.** Subscales and Composite Items of the HRQOL Questionnaire

Subscale	Items	No. of items	Cronbach α	Strength of internal reliability or comments
Activity limitations and life disruptions	Lack of energy, mobility problems, boredom, plans for the future, caring for others, education and work, change where you live	7	0.671	Moderate to acceptable
Worry about cancer and the future	Watch closely for new symptoms, worry about infections, worry about medication, worry about health in the future, worry about dying, worry about becoming a parent/having more children, worry about passing cancer on to the next generation	7	0.710	Acceptable
Self-esteem	Lack of self-confidence, loss of control, changes to appearance	3	0.765	Acceptable
Relationships	Isolation, people treating you differently, dependent on others, romantic relationships, sexual relationships	5	0.659	Moderate to acceptable
Positive changes	Mentally stronger, motivation to achieve goals, motivation to live life to the full, priorities	4	0.770	Acceptable

Abbreviation: HRQOL, health-related quality of life.

## Discussion

In this study, we conducted a robust and rigorous development of a novel HRQOL measurement tool designed specifically for AYAs with cancer. The experiences and opinions of 365 AYAs aged 14 to 39 years and 28 HCPs from 31 cancer centers across 20 countries shaped this pioneering work across the different phases. The final HRQOL questionnaire supplements the QLQ-C30 and includes 30 physical and psychosocial symptom and functioning questions of importance and relevance to this unique population. The content validity of the questionnaire is strengthened by our participatory research design, placing AYAs at the center of the development process, and our partnership with members of a cancer advisory panel of individuals aged 16 to 32 years.

More than half of the HRQOL questionnaire items are not included in existing EORTC QLG questionnaires, reiterating that AYAs are a special population with unique challenges and HRQOL concerns. Consistent with previous research,^23,24^ we highlighted that fertility and body image concerns are important areas to ask AYAs, yet only 24% of participants mentioned these areas spontaneously during the issue generation interviews. Thus, it underlines the importance of including such questions to prompt conversations. Previous research has shown that, after treatment, AYAs are often unclear about their fertility status.^25^ AYAs in our study stated that they had not considered fertility or the genetic risk of cancer before seeing the questionnaire and would subsequently check with their physicians. When asked whether these questions were upsetting, only 5 participants agreed. Questions about death and intimate relationships were seen as sensitive, particularly in certain cultures, but nonetheless valuable; the question on consequences for sexual relationships was the fifth highest-rated priority. Completion rates were high for all questions, including those about sexual and romantic relationships. We added a not-applicable response option for these items.

An expected adverse outcome of cancer in terms of loss of friendships was not obvious, and this question was removed following poor performance. AYAs explained that cancer made them aware of true friends and that relationships were maintained and strengthened through opportunities to connect via social media. Other changes in friendship networks (eg, forging new friendships with fellow patients) were also not evident but might be explained by the study coinciding with the lockdown restrictions of the COVID-19 pandemic.

Of the 30 questions included in the HRQOL questionnaire, 4 measure positive changes. Motivation to live life to the full and reorganization of priorities were among the strongest-performing questions, which supports our inclusion of such questions to provide full coverage of the experience of AYAs with cancer. Feedback from AYAs and our advisory panel suggested that these questions were welcomed and could be potentially therapeutic. Although the role of benefit finding as a stress buffer is evident within the AYA oncology literature,^26^ none of the measures used in this population include positive questions.^13^

The HRQOL questionnaire’s short recall time frame allows it to track HRQOL changes over time, supporting its use in trials, clinical research, and practice. This work, carried out on behalf of the EORTC, aligns with efforts of the PROMIS team, with both measurement initiatives offering complementary perspectives on assessing HRQOL of AYAs with cancer.^15,16^ Our planned international field study will validate the HRQOL questionnaire structure and confirm its acceptability among a broader group of AYAs.

### Limitations

This cross-cultural survey study captured the experiences of AYAs and HCPs from diverse backgrounds; however, it did not report response rates to the study invitation. Our future work will endeavor to record response rates. Furthermore, although the final HRQOL questionnaire is robust, it may not capture subtle differences in HRQOL across developmental subgroups or diverse cancer and treatment types. During the international validation study, the Write in Symptoms/Problems Scale^27^ will be included for AYAs to nominate and rate additional symptoms not covered by the HRQOL questionnaire. We also propose that the instrument could be supplemented with additional items from the EORTC QLG Item Library.^19^ Feedback from the pilot testing suggests that the measure already covers the broad spectrum of HRQOL concerns for AYAs. Adding questions to the 60 questions (30 each from the QLQ-C30 and the HRQOL questionnaire) should be carefully considered and justified.

## Conclusions

In this survey study involving AYAs and HCPs in 20 countries, we created a measurement tool capturing HRQOL issues of importance and relevance to this unique population of individuals aged 14 to 39 years with different cancer and treatment types across diverse cultures. The resulting HRQOL questionnaire holds promise as a reliable and valid tool for use in both clinical trials and practice.

## Notes

Supplement 1. **eAppendix 1.** Phase 1 Participant (14-39 years) Interview Schedule **eAppendix 2.** Phase 3 Participant (14-39 years) Interview Schedule **eTable 1.** Socio-Demographic and Clinical Characteristics of the Young Adults N=29 **eTable 2.** Decisions Made for Each HRQoL Issue Following Phase 1 Issue Generation and Review **eTable 3.** Characteristics of the HCPs Involved in Phase 1 **eTable 4.** Decisions for Each Item Following Phase 3 Pilot Testing

Supplement 2. **eTable 5.** Spearman Correlations Between AYA and QLQ-C30 Question Scores **eTable 6.** Correlations Between QLQ-AYA Provisional Questions

Supplement 3.
Nonauthor Collaborators

Supplement 4.
Data Sharing Statement

## References

[1] Smith AW, Seibel NL, Lewis DR, . Next steps for adolescent and young adult oncology workshop: an update on progress and recommendations for the future. Cancer. 2016;122(7):988-999. doi:10.1002/cncr.29870 26849003 PMC7521143

[2] Coccia PF, Altman J, Bhatia S, . Adolescent and young adult oncology: clinical practice guidelines in oncology. J Natl Compr Canc Netw. 2012;10(9):1112-1150. doi:10.6004/jnccn.2012.0117 22956810

[3] Arnett JJ, Žukauskienė R, Sugimura K. The new life stage of emerging adulthood at ages 18-29 years: implications for mental health. Lancet Psychiatry. 2014;1(7):569-576. doi:10.1016/S2215-0366(14)00080-7 26361316

[4] Fern LA, Whelan JS. Recruitment of adolescents and young adults to cancer clinical trials—international comparisons, barriers, and implications. Semin Oncol. 2010;37(2):e1-e8. doi:10.1053/j.seminoncol.2010.04.002 20494693

[5] Downs-Canner S, Shaw PH. A comparison of clinical trial enrollment between adolescent and young adult (AYA) oncology patients treated at affiliated adult and pediatric oncology centers. J Pediatr Hematol Oncol. 2009;31(12):927-929. doi:10.1097/MPH.0b013e3181b91180 19855302

[6] Bibby H, White V, Thompson K, Anazodo A. What are the unmet needs and care experiences of adolescents and young adults with cancer? a systematic review. J Adolesc Young Adult Oncol. 2017;6(1):6-30. doi:10.1089/jayao.2016.0012 27454408

[7] Berkman AM, Murphy KM, Siembida EJ, . Inclusion of patient-reported outcomes in adolescent and young adult phase III therapeutic trials: an analysis of cancer clinical trials registered on ClinicalTrials.gov. Value Health. 2021;24(12):1820-1827. doi:10.1016/j.jval.2021.06.012 34838280 PMC8630401

[8] Aaronson NK, Ahmedzai S, Bergman B, . The European Organization for Research and Treatment of Cancer QLQ-C30: a quality-of-life instrument for use in international clinical trials in oncology. J Natl Cancer Inst. 1993;85(5):365-376. doi:10.1093/jnci/85.5.365 8433390

[9] Varni JW, Burwinkle TM, Katz ER, Meeske K, Dickinson P. The PedsQL in pediatric cancer: reliability and validity of the Pediatric Quality of Life Inventory generic core scales, multidimensional fatigue scale, and cancer module. Cancer. 2002;94(7):2090-2106. doi:10.1002/cncr.10428 11932914

[10] Klassen AF, Strohm SJ, Maurice-Stam H, Grootenhuis MA. Quality of life questionnaires for children with cancer and childhood cancer survivors: a review of the development of available measures. Support Care Cancer. 2010;18(9):1207-1217. doi:10.1007/s00520-009-0751-y 19834745

[11] Siembida EJ, Reeve BB, Zebrack BJ, Snyder MA, Salsman JM. Measuring health-related quality of life in adolescent and young adult cancer survivors with the National Institutes of Health Patient-Reported Outcomes Measurement Information System: comparing adolescent, emerging adult, and young adult survivor perspectives. Psychooncology. 2021;30(3):303-311. doi:10.1002/pon.5577 33073416 PMC7965252

[12] Reeve BB, McFatrich M, Pinheiro LC, . Cognitive interview-based validation of the Patient-Reported Outcomes version of the Common Terminology Criteria for Adverse Events in adolescents with cancer. J Pain Symptom Manage. 2017;53(4):759-766. doi:10.1016/j.jpainsymman.2016.11.006 28062347 PMC5374011

[13] Sodergren SC, Husson O, Robinson J, ; EORTC Quality of Life Group. Systematic review of the health-related quality of life issues facing adolescents and young adults with cancer. Qual Life Res. 2017;26(7):1659-1672. doi:10.1007/s11136-017-1520-x 28251543 PMC5486886

[14] National Cancer Institute. Closing the gap: research and care imperatives for adolescents and young adults with cancer. 2006. U.S. Department of Health and Human Services, National Institutes of Health. Accessed May 24, 2024. https://www.cancer.gov/types/aya/research/ayao-august-2006.pdf.

[15] Salsman JM, Danhauer SC, Moore JB, . Optimizing the measurement of health-related quality of life in adolescents and young adults with cancer. Cancer. 2020;126(22):4818-4824. doi:10.1002/cncr.33155 32910454 PMC8005324

[16] Husson O, Sodergren SC, Darlington AS. The importance of a collaborative health-related quality of life measurement strategy for adolescents and young adults with cancer. Cancer. 2021;127(10):1712-1713. doi:10.1002/cncr.33416 33496340

[17] Wheelwright S, Bjordal K, Bottomley A, ; EORTC Quality of Life Group. EORTC Quality of Life Group Guidelines for Developing Questionnaire Modules. 5th ed. EORTC; 2021. Accessed September 25, 2024. https://www.eortc.org/app/uploads/sites/2/2022/07/Module-Guidelines-Version-5-FINAL.pdf

[18] Best practices for survey research. American Association for Public Opinion Research. Accessed September 12, 2025. https://aapor.org/standards-and-ethics/best-practices/#1668111466330-2e542b06-fd59.

[19] Braun V, Clarke V. Using thematic analysis in psychology. Qual Res Psychol. 2006;3(2):77-101. doi:10.1191/1478088706qp063oa

[20] Kulis D, Bottomley A, Whittaker C, . The use of the EORTC Item Library to supplement EORTC quality of life instruments. Value Health. 2017;20(9):A775. doi:10.1016/j.jval.2017.08.2236

[21] Sodergren SC, Husson O, Rohde GE, . A life put on pause: an exploration of the health-related quality of life issues relevant to adolescents and young adults with cancer. J Adolesc Young Adult Oncol. 2018;7(4):453-464. doi:10.1089/jayao.2017.0110 29565709

[22] Sodergren SC, Husson O, Rohde GE, ; EORTC Quality of Life Group. Does age matter? a comparison of health-related quality of life issues of adolescents and young adults with cancer. Eur J Cancer Care (Engl). 2018;27(6):e12980. doi:10.1111/ecc.12980 30485601

[23] Benedict C, Shuk E, Ford JS. Fertility issues in adolescent and young adult cancer survivors. J Adolesc Young Adult Oncol. 2016;5(1):48-57. doi:10.1089/jayao.2015.0024 26812452 PMC4779291

[24] Wytiaz V, Jackson Levin N, Tan CY, . Body image disturbances in adolescent and young adult cancer patients confronting infertility risk and fertility preservation decisions. J Psychosoc Oncol. 2024;42(2):208-222. doi:10.1080/07347332.2023.2235607 37452662 PMC10788379

[25] Wright CI, Coad J, Morgan S, Stark D, Cable M. ‘Just in case’: the fertility information needs of teenagers and young adults with cancer. Eur J Cancer Care (Engl). 2014;23(2):189-198. doi:10.1111/ecc.12137 24138775

[26] Rosenberg AR, Bradford MC, Barton KS, . Hope and benefit finding: results from the PRISM randomized controlled trial. Pediatr Blood Cancer. 2019;66(1):e27485. doi:10.1002/pbc.27485 30270489 PMC6249081

[27] Rojas-Concha L, Arrarrás JI, Conroy T, ; on behalf the EORTC Quality of Life Group. Acceptability and usefulness of the EORTC ‘Write In three Symptoms/Problems’ (WISP): a brief open-ended instrument for symptom assessment in cancer patients. Health Qual Life Outcomes. 2024;22(1):28. doi:10.1186/s12955-024-02244-z 38532393 PMC10964595

