# Supplementary material for: Development of a Health-Related Quality of Life Tool for Adolescents and Young Adults With Cancer
Source: JAMA Netw Open. 2025 Dec 19;8(12):e2549071. doi: 10.1001/jamanetworkopen.2025.49071 (PMC12717613; doi:10.1001/jamanetworkopen.2025.49071)
Supplement: Supplement 1. — eAppendix 1. Phase 1 Participant (14-39 years) Interview Schedule eAppendix 2. Phase 3 Participant (14-39 years) Interview Schedule eTable 1. Socio-Demographic and Clinical Characteristics of the Young Adults N=29 eTable 2. Decisions Made for Each HRQoL Issue Following Phase 1 Issue Generation and Review eTable 3. Characteristics of the HCPs Involved in Phase 1 eTable 4. Decisions for Each Item Following Phase 3 Pilot Testing [file jamanetwopen-e2549071-s001.pdf]

Supplemental Online Content

Sodergren SC, Husson O, Janssen S, et al; on behalf of the EORTC Quality of Life Group. Development of a health-related quality of life tool for adolescents and young adults with cancer. *JAMA Netw Open*. 2025;8(12):e2549071. doi:10.1001/jamanetworkopen.2025.49071

eAppendix 1. Phase 1 Participant (14-39 years) Interview Schedule

eAppendix 2. Phase 3 Participant (14-39 years) Interview Schedule

eTable 1. Socio-Demographic and Clinical Characteristics of the Young Adults N=29

eTable 2. Decisions Made for Each HRQoL Issue Following Phase 1 Issue Generation and Review

eTable 3. Characteristics of the HCPs Involved in Phase 1

eTable 4. Decisions for Each Item Following Phase 3 Pilot Testing

This supplemental material has been provided by the authors to give readers additional information about their work.

© 2025 Sodergren SC et al. *JAMA Network Open*

eAppendix 1. Phase 1 Participant (14-39 years) Interview Schedule

Phase 1 (a) Issue Generation

1. Introductory remarks to explain the nature and purpose of the study.

We are asking for your help with our study looking at how cancer and its treatment can affect the lives of teenagers and young adults like yourself. First of all, we would like to collect some information about you.

Complete the CRF as much as possible explaining that, with their permission and as indicated on the consent form, we will look through their medical notes to find out details about their cancer and treatments.

2. General open-ended question

Please could you tell me about all the different ways in which your life has been affected by cancer and its treatment.

3. Use probes to elicit more information.

In what other ways has your life been affected?

How did you deal with that? Would you like to tell me more about that?

Other young people have mentioned things like…… (friendships, education, having to rely on others, plans for the future). Have any of these things been affected for you?

4. Show the patient the EORTC QLQ-C30 to prompt or stimulate further suggestions.

Here is an existing questionnaire that asks about you and the problems you might have faced. This questionnaire is a general cancer one designed for older people. I would like you to read through this questionnaire and see if there is anything you think is important to you. You do not need to complete the questionnaire.

Which of the questions on this questionnaire would you say are the most important to you?

Do you think this questionnaire has overlooked anything important?

5. Summarise the issues raised during the interview and identify which issues are the most important.

We have discussed a number of things. These are…

Which of these things do you think are the most important?

Thank you for your help.

© 2025 Sodergren SC et al. *JAMA Network Open*

eAppendix 2. Phase 3 Participant (14-39 years) Interview Schedule

We are asking for your help in developing a new questionnaire to measure how cancer and its treatment affect young people's quality of life. Firstly, I would like to ask you some questions about yourself, your cancer and its treatment. Don't worry if you don't remember all the details because, with your permission, we will look at your medical notes to complete any gaps.

Completion of the CRF (as much as possible)

We have two questionnaires that ask about you and your health and quality of life. We would like you to complete these questionnaires. After you have completed them, I will ask you whether you think we have asked the right questions in the right way. We want to be sure that we cover the most important aspects of patients' experience of cancer and its treatment.

EORTC QLQ-C30
Place EORTC QLQ-C30 before the patient who then completes it with help from the researcher if necessary.

Provisional EORTC Adolescent and Young Adult Questionnaire
As a result of your cancer and its treatment you may have experiences in common with other young people who have also had cancer. These particular experiences might not covered be by this more general questionnaire. We would like to add some extra questions to take into account those things which may be important to you and other young people. We are now asking for your help in devising these additional questions.

We think that this questionnaire may be more useful for younger people.

Place the provisional EORTC Adolescent and Young Adult Quality of Life Questionnaire before the patient who completes it with help from the researcher if necessary

<u>Relevance and Importance Ratings / Comments (see below)</u>
I would now like to go through the questions on the adolescent and young adult questionnaire and ask you to indicate for each question whether it is relevant to you , i.e., whether you think the issue is something you have experienced at some time and how important/troublesome it was ranging from 1 “Not at all” to 4 “Very much”. As you go through the questions, let me know any thoughts you have. For example, are any of the questions difficult, annoying, confusing, upsetting or intrusive?

Place the provisional EORTC Adolescent and Young Adult Quality of Life Questionnaire (Relevance and importance ratings) before the patient who completes it with help from the researcher if necessary.

Make a note of any comments made about any issue or any general issues on the comment sheet. Encourage the patient to “think aloud”.

Completion of the interview
You identified issues that you felt were not relevant to you such as…….. Were there any other questions in either questionnaire that you feel are irrelevant or not important to you?

Can you think of additional issues that are relevant / important for you but are not included in either questionnaire?

Of all the issues we have talked through, which ones would you say must be included in a questionnaire for adolescents and young people with cancer, which ones would you consider to be priority issues?
Is there anything else you wish to add?

Record any suggestions.

Thank you for your time and help.

© 2025 Sodergren SC et al. *JAMA Network Open*

Relevance and Importance Ratings

Please circle the response that reflects your experience in terms of relevance: Is this something you have experienced since your diagnosis (yes or no) and importance: To what extent this issue has troubled or bothered you (ranging from "not at all" to "very much")

No.	Items	RELEVANT?		IF RELEVANT, HOW IMPORTANT?				Comments
				Not at all	A little	Quite a bit	Very much	
1.	Have you lacked energy?	YES	NO	1	2	3	4	
2.	Have you been watching yourself closely for any new symptoms of disease?	YES	NO	1	2	3	4	
3.	Have you had problems with mobility?	YES	NO	1	2	3	4	
4.	Have you had problems taking part in social activities	YES	NO	1	2	3	4	
5.	Have you felt isolated from your friends?	YES	NO	1	2	3	4	
6.	Have you worried that people are treating you differently?	YES	NO	1	2	3	4	
7.	Have you felt bored?	YES	NO	1	2	3	4	
8.	Have you felt stressed?	YES	NO	1	2	3	4	
9.	Have you spent time thinking about your disease?	YES	NO	1	2	3	4	

© 2025 Sodergren SC et al. *JAMA Network Open*

10.	Have you worried about your health in the future?	YES	NO	1	2	3	4	
11.	Have you worried about dying?	YES	NO	1	2	3	4	
12.	Have you worried about getting infections?	YES	NO	1	2	3	4	
13.	Have you felt restricted in the types of food and drink you can have?	YES	NO	1	2	3	4	
14.	Has your family been negatively affected?	YES	NO	1	2	3	4	
15.	Have your friends been negatively affected?	YES	NO	1	2	3	4	
16.	Have you worried you might have to change your career plans?	YES	NO	1	2	3	4	
17.	Have you had problems making plans for the future?	YES	NO	1	2	3	4	
18.	Have you been dependent on others?	YES	NO	1	2	3	4	
19.	Have you lost friendships?	YES	NO	1	2	3	4	
20.	Has your romantic life been negatively affected?	YES	NO	1	2	3	4	
21.	Has your sex life been negatively affected?	YES	NO	1	2	3	4	
22.	Have you worried about changes to your appearance?	YES	NO	1	2	3	4	

© 2025 Sodergren SC et al. *JAMA Network Open*

23.	Have you lacked self-confidence?	YES	NO	1	2	3	4	
24.	Have you felt that you have lost control over your life?	YES	NO	1	2	3	4	
25.	Have you become more negative about life?	YES	NO	1	2	3	4	
26.	Have you worried about your disease or treatment causing you health problems in the future?	YES	NO	1	2	3	4	
27.	Have you been worried about your ability to have children?	YES	NO	1	2	3	4	
28.	Have you worried about passing cancer on to the next generation?	YES	NO	1	2	3	4	
29.	Have you felt that it is unfair that you became ill?	YES	NO	1	2	3	4	
30.	Has your relationship with any of your family members improved?	YES	NO	1	2	3	4	
31.	Has your relationship with any of your friends improved?	YES	NO	1	2	3	4	
32.	Have you made new friends?	YES	NO	1	2	3	4	
33.	Have you become more confident?	YES	NO	1	2	3	4	
34.	Have you become mentally stronger?	YES	NO	1	2	3	4	
35.	Have you felt more mature?	YES	NO	1	2	3	4	

© 2025 Sodergren SC et al. *JAMA Network Open*

36.	Have you made positive lifestyle changes (e.g., healthy eating, exercise)?	YES	NO	1	2	3	4	
37.	Have you become more positive about life?	YES	NO	1	2	3	4	
38.	Have you felt more motivated to live life to the full?	YES	NO	1	2	3	4	
39.	Have you felt more motivated to achieve your personal goals?	YES	NO	1	2	3	4	
40.	Has your experience helped you to distinguish between important and non-important things in life?	YES	NO	1	2	3	4	
41.	Problem with weight gain	YES	NO	1	2	3	4	
42.	Problem with having lost weight	YES	NO	1	2	3	4	
43.	Worry about ability to care for others	YES	NO	1	2	3	4	
44.	Problem with having to change where you live	YES	NO	1	2	3	4	
45.	Worry about impact on education	YES	NO	1	2	3	4	
46.	Worry about impact on job	YES	NO	1	2	3	4	
47.	Problem having to take regular medication	YES	NO	1	2	3	4	
48.	Have your religious or spiritual beliefs weakened?	YES	NO	1	2	3	4	

© 2025 Sodergren SC et al. *JAMA Network Open*

49.	Have your religious or spiritual beliefs strengthened?	YES NO	1 2 3 4	
50.	Women only: Problem with early menopause	YES NO	1 2 3 4	

© 2025 Sodergren SC et al. *JAMA Network Open*

eTable 1. Socio-demographic and clinical characteristics of the young adults N=29

**Variable**	Young Adults (N=29)
Participants recruited per country	
Denmark	5 (17%)
Germany	4 (14%)
Jordan	10 (32%)
Netherlands	1 (3%)
Turkey	4 (14%)
UK	5 (17%)
Gender	
Male	10 (34%)
Female	19 (66%)
Age (years)	
Mean (standard deviation)	31.6 (4.4)
Missing	1 (3%)
Ethnicity	
White	18 (62%)
Arab	10 (34%)
Mixed	1 (3%)
Education level	
Less than compulsory school education	2 (7%)
Compulsory school education	4 (14%)
Post compulsory school education (college)	5 (17%)
University	16 (55%)
Missing	2 (7%)
Employment status	
Full time	8 (28%)
Part time	5 (17%)
Homemaker	1 (3%)
Sick leave	9 (31%)
None	6 (21%)
Missing	1 (3%)
Living situation	
Alone	4 (14%)
Parents	6 (21%)
Partner	16 (55%)
Living with others	2 (7%)
Missing	1 (3%)
Time since diagnosis (months)	
Median	6
Range	1-108
Missing	1 (3%)
Disease status	
Localised	14 (48%)
Metastatic	10 (34%)
Not applicable (Leukaemia, lymphoma)	4 (14%)
Missing	1 (3%)
Diagnosis[1]	
Breast	8 (28%)
Lymphoma	4 (14%)
Testicular	4 (14%)
Gynaecological	3 (10%)
Colorectal	3 (10%)
Leukaemia	2 (7%)
Sarcoma	2 (7%)
Liver	2 (7%)

© 2025 Sodergren SC et al. *JAMA Network Open*

Brain	1 (3%)
Bone	1 (3%)
Lung	1 (3%)
Appendix	1 (3%)
Gastric	1 (3%)
Myelodysplastic Syndrome	1 (3%)
Treatment status	
Currently on treatment	23 (79%)
Supportive / palliative care	5 (17%)
Missing	1 (3%)
Treatment intent	
Curative	26 (90%)
Palliative	2 (7%)
Missing	1 (3%)
Treatment type[2]	
Chemotherapy	25 (86%)
Targeted therapy	2 (7%)
Hormonal	2 (7%)
Radiotherapy	1 (3%)
Immunotherapy	1(3%)
Other (not specified)	1 (3%)
Missing	3 (10%)
Co-morbidities[3]	
None	15 (52%)
One or more	12 (41%)
Gynaecological	2 (7%)
Rheumatic	2 (7%)
Allergies	1 (3%)
Depression	1 (3%)
Epilepsy	1 (3%)
Fibromyalgia	1 (3%)
Obesity	1 (3%)
Pulmonary embolism	1 (3%)
Respiratory	1 (3%)
Skin problems	1 (3%)
Thrombosis	1 (3%)
Missing	2 (7%)
ECOG Performance Status	
0 (Fully active)	13 (41%)
1 (Restricted in physical strenuous activity)	12 (41%)
2 (Unable to carry out work activities)	2 (7%)
3 (Limited self-care)	0
Missing	2 (7%)

[1] For four patients, cancers were recorded at more than one site

[2]Patients indicated all current treatment types

[3]Several patients presented more than one co-morbidity

© 2025 Sodergren SC et al. *JAMA Network Open*

eTable 2. Decisions made for each HRQoL issue following Phase 1 issue generation and review

Issue	Decision	Reason	Question selected from the Item Library	New question created
**1. Greater awareness of physical symptoms**	Retain	N/A	Have you been watching yourself closely for any new symptoms?	N/A
**2. Pain**	Remove	Redundancy with QLQ-C30	N/A	N/A
**3. Nausea**	Remove	Redundancy with QLQ-C30	N/A	N/A
**4. Vomiting**	Remove	Redundancy with QLQ-C30	N/A	N/A
**5. Tiredness**	Remove	Redundancy with QLQ-C30	N/A	N/A
**6. Lack of energy**	Retain	N/A	Have you lacked energy?	N/A
**7. Loss of strength**	Remove	Redundancy with QLQ-C30	N/A	N/A
**8. Shortness of breath**	Remove	Redundancy with QLQ-C30	N/A	N/A
**9. Trouble sleeping**	Remove	Redundancy with QLQ-C30	N/A	N/A
**10. Difficulty with weight**	Split into two questions (weight gain and weight loss)	N/A	Has weight gain been a problem for you?"<br>Has weight loss been a problem for you?	N/A
**11. Mobility problems**	Retain	N/A	Have you had problems with mobility?	N/A
**12. Difficulty concentrating**	Remove	Redundancy with QLQ-C30	N/A	N/A
**13. Impact on hobbies or leisure time activities**	Remove	Redundancy with QLQ-C30	N/A	N/A
**14. Not able to bathe, take showers or dress yourself**	Remove	Redundancy with QLQ-C30	N/A	N/A
**15. Not able to go out**	Retain	N/A	No	Have you had problems taking part in social activities (e.g., meeting friends or family)?

**16. Education has been interrupted**	Retain-Conditional	N/A	No	Preceded by a conditional question: Are you in education?<br>If yes, have you worried about the impact of your disease or treatment<br>on your education?
**17. Changed career plans**	Retain	N/A	No	Have had to change your career plans?
**18. Not able to work**	Retain-Conditional	N/A	No	Preceded by a conditional question: Do you have a job (paid or voluntary)? If yes, have you worried about the impact of your disease or treatment<br>on your work?
**19. Fallen behind where you want to be in life**	Remove	Identified as confusing	N/A	N/A
**20. Loss of friends**	Retain	N/A	No	Have you lost friends because of your disease or treatment?
**21. Isolation from friends**	Retain	N/A	Have you felt isolated from those close to you (e.g., family, friends)? Modified to: Have you felt isolated from your friends because of your disease or treatment?	N/A
**22. Less time to go out with friends**	Remove	Overlap with AYA issue 15	N/A	N/A
**23. Impact on romantic / sexual relationships**	Retain- Spilt into two questions (romantic life and sex life)	N/A	Has the disease or treatment affected your sex life (for the worse)? Modified to: Has your sex life been affected?	Has your romantic life been affected?
**24. Strengthening existing relationships with friends and family**	Retain- Spilt into two questions (family and friends)	N/A	Are your relationships with family and/or friends stronger? Modified to: Has your relationship with any of your family members improved?	N/A

			Has your relationship with any of your friends improved?	
**25. Opportunity to make new friends**	Retain	N/A	No	Have you made new friends?
**26. Greater dependence on others**	Retain	N/A	Have you worried about becoming dependent on others? Modified to: Have you been dependent on others?	N/A
**27. Greater burden on others**	Remove	Overlap with AYA issue 26. Identified as upsetting	N/A	N/A
**28. Change in living situation as a result of the diagnosis, e.g., move back to parent's home**	Retain-Conditional	N/A	No	Preceded by a conditional question: Have you changed where you live because of your disease or treatment? If yes, has it been a problem for you?
**29. Impact on family and friends**	Retain- Split into two separate questions (family and friends)	N/A	No	Has your family been negatively affected?<br>Have your friends been negatively affected?
**30. Impact on family life**	Remove	Overlap with AYA issue 29	N/A	N/A
**31. Unable to care for others**	Retain-Conditional	N/A	No	Preceded by a conditional question:<br>Do you have caring responsibilities for others?<br>Are you unable to care for other people? If yes, have you worried about your ability to care for others because of your disease or treatment?
**32. Less tolerant of others**	Remove	Measuring something conceptually different (not HRQoL)	N/A	N/A
**33. More sympathetic to others**	Remove	Measuring something conceptually	N/A	N/A

		different (not HRQoL)		
**34. Boredom**	Retain	N/A	No	Have you felt bored?
**35. Depression**	Remove	Redundancy with QLQ-C30	N/A	N/A
**36. Anxiety**	Remove	Redundancy with QLQ-C30	N/A	N/A
**37. Preoccupation with illness**	Retain	N/A	No	Have you spent time thinking about your illness?
**38. Embarrassment**	Remove	Overlap with AYA issue 47	N/A	N/A
**39. Fear of recurrence**	Remove	Overlap with AYA issue 40	N/A	N/A
**40. Worry about what the future holds**	Retain-Spilt into two separate questions to measure late effects of cancer and treatment and cancer recurrence	N/A	Have you worried about your treatment causing future health problems? Modified to: Have you worried about your disease or treatment causing you health problems in the future? Have you worried about your future health? Modified to: Have you worried about your health in the future?	N/A
**41. Worry about dying**	Retain	N/A	Have you worried about dying?	N/A
**42. Shock of diagnosis**	Remove	Anticipated ceiling effect	N/A	N/A
**43. Anger**	Remove	Overlap with AYA issue 44.	N/A	N/A
**44. Question why is this happening to me?**	Retain	N/A	I have felt that it is unfair that I am ill. Modified to: Have you felt that it is unfair that you became ill?	N/A
**45. Feel let down by your body**	Remove	Overlap with AYA issue 44. Identified as confusing	N/A	N/A
**46. Altered appearance**	Retain	N/A	No	Have you worried about changes to your appearance?
**47. More self-conscious and concern about one's appearance**	Remove	Overlap with AYA issue 46.	N/A	N/A

© 2025 Sodergren SC et al. *JAMA Network Open*

**48. Less concerned about one's appearance**	Remove	Measuring something conceptually different (not HRQoL)	N/A	N/A
**49. Greater self-awareness**	Remove	Identified as confusing	N/A	N/A
**50. Stronger person**	Retain	N/A	No	Have you become mentally stronger?
**51. Better person**	Remove	Measuring something conceptually different (not HRQoL)	N/A	N/A
**52. Braver**	Remove	Overlap with AYA issue 50. Identified as upsetting	N/A	N/A
**53. Increased maturity**	Retain	N/A	No	Have you felt more mature?
**54. Greater confidence**	Retain	N/A	No	Have you become more confident?
**55. Lowered self-confidence**	Retain	N/A	Have you lacked self-confidence?	N/A
**56. Changed outlook on life**	Retain- Split into two separate questions to measure positive and negative outlook	N/A	Have you had a positive outlook on life in the last week? Modified to: Have you become more positive about life?	Have you become more negative about life?
**57. More positive outlook on life**	Retain	N/A	As above	N/A
**58. Different priorities in life**	Retain	N/A	Has your experience helped you to distinguish between important and non-important things in life?	N/A
**59. Greater desire to live life to the fullest**	Retain	N/A	Have you felt more motivated to live life to the full?	N/A
**60. Greater life experience**	Remove	Overlap with AYA issue 59. Identified as confusing	N/A	N/A
**61. Greater awareness of one's mortality**	Remove	Overlap with AYA issue 41.	N/A	N/A
**62. Inability to plan for the future**	Retain		No	Have you had problems making plans for the future?
**63. Greater motivation to succeed academically**	Remove	Overlap with AYA issue 64	N/A	N/A

**64. Greater motivation to achieve personal goals**	Retain	N/A	No	Have you felt more motivated to achieve your personal goals?
**65. Motivation to lead a healthier lifestyle**	Retain	N/A	No	Have you made positive lifestyle changes?
**66. Take greater care to avoid infections**	Retain	N/A	Have you worried about picking up an infection? Modified to: Have you worried about getting infections?	N/A
**67. Difficulty adjusting to being ill and having to take medication**	Retain-Conditional	N/A	No	Preceded by a conditional question: Do you have to take regular medication because of your disease or treatment? If yes, has it been a problem for you?
**68. Restricted food choice**	Retain and combine with AYA issue 69	N/A	Were you restricted in the types of food you can eat as a result of your disease or treatment? Modified to: Have you felt restricted in the types of food and drink you can have?	N/A
**69. Restricted choice of drinks**	Retain and combine – see above	N/A	As above	N/A
**70. Dissatisfaction with care**	Remove	Measuring something conceptually different (not HRQoL)	N/A	N/A
**71. Lack of age-appropriate information**	Remove	Measuring something conceptually different (not HRQoL)	N/A	N/A
**72. Treatment burden**	Remove	Identified as confusing	N/A	N/A
**73. Difficulty readjusting to life after treatment is over**	Remove	Irrelevant to AYAs on treatment	N/A	N/A
**74. Concern over long-term effects of disease or treatment**	Remove	Overlap with AYA issue 40	N/A	N/A
**75. Concerns over fertility**	Retain	N/A	Have you been concerned about your ability to	N/A

			have children? Modified to: Have you worried about your ability to have children?	
**76. Desire for life to return to "normal"**	Remove	Measuring something conceptually different (not HRQoL)	N/A	N/A
**77. Financial difficulties**	Remove	Redundancy with QLQ-C30	N/A	N/A
**Additional Issues generated from the young adults**				
**Early Menopause**	Retain-Conditional	N/A	No	Preceded by a conditional question: Have you experienced early menopause because of your disease or treatment? If yes, has it been a problem for you?
**Loss of control**	Retain		Have you been afraid of losing control? Modify to: Have you felt that you have lost control of your life?	N/A
**Mood swings**	Remove	Overlap with AYA issues and QLQ-C30.	N/A	N/A
**Stressed**	Retain	N/A	Have you felt stressed?	N/A
**Guilt**	Remove	Overlap with AYA issue 27.	N/A	N/A
**Live life at a slower pace**	Remove	Identified as difficult to interpret – can be both positive and negative.	N/A	N/A
**Sense of identity**	Remove	Overlap with AYA issue 49 which was removed,	N/A	N/A
**Treated differently by others**	Retain	N/A	No	Have you worried that people are treating you differently?
**Concern over passing on cancer to future generations**	Retain	N/A	No	Have you worried about passing

© 2025 Sodergren SC et al. *JAMA Network Open*

				cancer on to the next generation?
**Spiritual or religious faith (strengthened and weakened)**	Retain-Split into two separate questions (strengthened and weakened). Conditional	N/A	Has your religious faith or your sense of spirituality strengthened? Modified to: (Preceded by a conditional question: Do you have any religious or spiritual beliefs? If yes,) Have your religious or spiritual beliefs strengthened as a result of your disease or treatment?	Have your religious or spiritual beliefs weakened as a result of your disease or treatment?

N/A refers to not applicable

eTable 3. Characteristics of the HCPs involved in Phase 1

	**Phase 1a interviews** (N=20)	**Phase 1b Issue rating** (N=8)
Patients recruited per country (country Identifier)		
Denmark	3 (15%)	
Germany	3 (15%)	
Jordan	6 (30%)	
Poland	0	
Switzerland	3 (15%)	
The Netherlands	0	
Turkey	2 (10%)	
UK	3 (15%)	
Gender		
Male	6 (30%)	
Female	14 (70%)	
Age (years)		
Mean (standard deviation)	39.4 (6.9)	
Range	29-52	
Specialist discipline		
Clinical oncology	2 (10%)	
Medical oncology	8 (40%)	
Nursing	5 (25%)	
Psychologist	2 (10%)	
Surgery	1 (5%)	
Support worker	1 (5%)	
Years involved in AYA care		
<1 year	0	
1-5 years	5 (25%)	
5-10 years	8 (40%)	
>10 years	6 (30%)	
Missing	1 (5%)	

© 2025 Sodergren SC et al. *JAMA Network Open*

eTable 4. Decisions for each item following Phase 3 pilot testing

	Question	Decision	Justification
1	Have you lacked energy?	Retain	Acceptable performance. Query raised by participants and collaborators about whether we need to distinguish between mental and physical energy. Consensus that it would be well understood. We do not wish to distinguish between types of energy and want to avoid creating an additional item.
2	Have you been watching yourself closely for any new symptoms of disease?	Retain	Acceptable performance.<br>Replace disease with cancer
3	Have you had mobility problems?	Retain	Acceptable performance
4	Have you had problems taking part in social activities	Remove	Redundancy with existing QLQ-C30 items: interference with social activities and leisure activities. Overlap with the social isolation question.
5	Have you felt isolated from your friends?	Re-word to "Have you felt isolated from those close to you (e.g., family, friends)?"	Acceptable performance. Isolation in general was identified as an omission.
6	Have you worried about people treating you differently?	Retain	Acceptable performance
7	Have you felt bored?	Retain	Acceptable performance
8	Have you felt stressed?	Remove	Correlated with QLQ-C30 items of tense, worry and irritable suggesting redundancy. Collaborator feedback implied translation issues and lack of clarity regarding the meaning of "stress"
9	Have you spent time thinking about your disease?	Remove	Lack of clarity. Feedback from participants about how to quantify the amount of time and that most people will think about their disease to some extent. Query from collaborators as to whether this relates to thinking about or worrying about disease.
10	Have you worried about your health in the future?	Retain	This question is retained in favour over question 26 (health problems in the future) due to its clarity and that it relates to late effects as well as cancer recurrence.
11	Have you worried about dying?	Retain	Highlighted as potentially sensitive but regarded as important to ask by both participants and collaborators. Except for the upsetting / inappropriate criterion, this question performed well.
12	Have you worried about getting infections?	Retain	Acceptable performance
13	Have you felt restricted in the types of food	Retain	Acceptable performance

© 2025 Sodergren SC et al. *JAMA Network Open*

	and drink you can have?		
14	Has your family been negatively affected?	Remove	Experienced as confusing and lack of knowledge as to how family members are feeling. Overlap with the QLQ-C30 question about interference with family life.
15	Have your friends been negatively affected?	Remove	Experienced as confusing and lack of knowledge as to how friends are feeling.
16	Have you had to change your career plans?	Remove	The direction of change is not known with this question. This question is covered by questions on employment and education.
17	Have you had problems making plans for the future?	Retain	Acceptable performance
18	Have you been dependent on others?	Re-word to "Have you worried about being dependent on others?"	Feedback suggested a need for clarity regarding the negative orientation of this question.
19	Have you lost friendships?	Remove	Poor performance on four of the decision rule criteria
20	Has your romantic life been negatively affected?	Retain	Addition of a not applicable response option. Although identified as upsetting / inappropriate and not relevant to all participants, it was regarded by AYAs and collaborators as important to include.
21	Has your sex life been negatively affected?	Retain	Addition of a not applicable response option. Although identified as upsetting / inappropriate and not relevant to all participants, it was regarded by AYAs and collaborators as important to include.
22	Have you worried about changes to your appearance?	Retain	Acceptable performance
23	Have you lacked self-confidence?	Retain	Acceptable performance. Move position so that it does not immediately follow the question about changes to appearance.
24	Have you felt that you have lost control over your life?	Retain	Acceptable performance
25	Have you become more negative about life?	Remove	Feedback from collaborators that this might not be measuring HRQoL and is more existential in content.
26	Have you worried about your disease or treatment causing you health problems in the future?	Remove	See comments relating to question 10 with which this question correlates.
27	Have you been worried about your ability to have children?	Re-word to "Have you worried about being able to	Acceptable performance. Re-worded on the recommendation of a male participant who felt that the question was only applicable to women. The revised version is applicable for all participants to answer and relates to AYAs who have already had children and might

		become a parent or have more children?”	want to have more as well as those who have not yet had a child.
28	Have you worried about passing cancer on to the next generation?	Retain	Acceptable performance. Further testing with members of a young people’s advisory panel supported the inclusion and acceptability of this question and its wording.
29	Have you felt that it is unfair that you became ill?	Remove	Feedback from collaborators that this question does not contribute to our understanding of HRQoL.
30	Has your relationship with any of your family members improved?	Remove	Feedback that this should not be the focus of the questionnaire
31	Has your relationship with any of your friends improved?	Remove	Feedback that this should not be the focus of the questionnaire
32	Have you made new friends?	Remove	Poor performance
33	Have you become more confident?	Remove	Lack of clarity as to what areas of life this question relates to. Overlap with mentally stronger.
34	Have you become mentally stronger?	Retain	Acceptable performance
35	Have you felt more mature?	Remove	Remove in favour of keeping the question about becoming mentally stronger.
36	Have you made positive lifestyle changes (e.g., healthy eating, exercise)?	Remove	Feedback from collaborators that this measures the complex issue of behaviour change rather than HRQoL.
37	Have you become more positive about life?	Remove	Feedback from collaborators that this might not be measuring HRQoL and is more existential in content.
38	Have you felt more motivated to live life to the full?	Retain	Although there is overlap with motivation to achieve personal goals, further testing with young people with cancer advisors and subsequent expert review emphasised that these two questions are measuring conceptually different constructs.
39	Have you felt more motivated to achieve your personal goals?	Retain	Acceptable performance
40	Has your experience helped you to distinguish between important and	Retain	Acceptable performance.

© 2025 Sodergren SC et al. *JAMA Network Open*

	non-important things in life?		
41	Problem with weight gain	Retain	Acceptable performance. Remove the screening part of the question so that all participants can answer it regardless of weight gain. Add a “not applicable” response option for respondents not experiencing weight gain.
42	Problem with having lost weight	Retain	Acceptable performance. Remove the screening part of the question so that all participants can answer it regardless of weight loss. Add a “not applicable” response option for respondents not experiencing weight loss.
43	Worry about ability to care for others	Retain	Acceptable performance. Include a “not applicable” response option and therefore remove the need for a screening question.
44	Problem with having to change where you live	Retain	Acceptable performance. Include a “not applicable” response option and therefore remove the need for a screening question.
45	Worry about impact on education	Combine with 46. Reword to: “Have you had problems with your education or work?”	Combine with impact on employment so that the question can be potentially answered by all participants and remove the need for a screening question. A “not applicable” option was added.
46	Worry about impact on job	Combine with 45	Combine with impact on education so that the question can be potentially answered by all participants and remove the need for a screening question. A “not applicable” option was added.
47	Problem having to take regular medication	Re-worded to “Have you been worried about having to take medication for the rest of your life”	Feedback that this question was vague in terms of what it is measuring.
48	Have your religious or spiritual beliefs weakened?	Remove	Poor performance. Feedback from collaborators that this might not be measuring HRQoL and is more existential in content. Feedback from participants that this might be a sensitive issue to ask about and that there might be a fear of disclosure in terms of weakening / loss of faith.
49	Have your religious or spiritual beliefs strengthened?	Remove	Feedback from collaborators that this might not be measuring HRQoL and is more existential in content.
50	Women only: Problem with early menopause	Retain	Acceptable performance. Change response options to include a not applicable option therefore removing the need for a screening question and for gendered language.
