# Supplementary material for: Development of a Health-Related Quality of Life Tool for Adolescents and Young Adults With Cancer
Source: JAMA Netw Open. 2025 Dec 19;8(12):e2549071. doi: 10.1001/jamanetworkopen.2025.49071 (PMC12717613; doi:10.1001/jamanetworkopen.2025.49071)
Supplement: Supplement 3. — Nonauthor Collaborators [file jamanetwopen-e2549071-s003.pdf]

Supplemental Online Content: Nonauthor Collaborators
*First name, last name, and suffix (if applicable) are required and will appear in PubMed.

*Group Name(s): EORTC Quality of Life Group

***First Name and Middle Initial(s)**	***Last Name**	***Suffix (eg, Jr, III)**	Academic Degrees	Institution	Location (city, state/province, country)	Role or Contribution, eg, chair, principal investigator	Group (if more than 1 Group listed in the byline) and/or Subgroup (eg, Steering Committee)
Tayhla	Ryder		BSocSi, Mres	Canteen	Sydney	Data collection and recruitment	
Oana C.	Lindner		Phd, MSc, BSc	University of Leeds	Leeds, UK	Study planning and local delivery	
Faatimah	Patel		RGN, BSc, BSc	Leeds Teaching Hospitals NHS Trust	Leeds, UK	Data collection and recruitment	
Denise	Hancock		RGN, BSc, PGD, PG Cert	Leeds Teaching Hospitals NHS Trust	Leeds, UK	Study planning and local delivery	
Ingunn	Holtskog		RN, MSc	Sorlandet Hospital	Kristiansand, Norway	Recruitment and clinical data	
Mor	Bar-Ilan		DMD	Sheba Medical Center	Tel Aviv, Israel	Data collection and recruitment	
