# Supplementary material for: Development of a Health-Related Quality of Life Tool for Adolescents and Young Adults With Cancer
Source: JAMA Netw Open. 2025 Dec 19;8(12):e2549071. doi: 10.1001/jamanetworkopen.2025.49071 (PMC12717613; doi:10.1001/jamanetworkopen.2025.49071)
Supplement: Supplement 4. — Data Sharing Statement [file jamanetwopen-e2549071-s004.pdf]

Data Sharing Statement

Sodergren. Development of a Health-Related Quality of Life Tool for Adolescents and Young Adults With Cancer. *JAMA Netw Open*. Published December 19, 2025.
doi:10.1001/jamanetworkopen.2025.49071

Data
Data available: Yes
Data types: Deidentified participant data
How to access data: S.C.Sodergren@soton.ac.uk
When available: With publication

Supporting Documents
Document types: None

Additional Information
Who can access the data: Researchers who have been approved by the EORTC Quality of Life Group
Types of analyses: Any purpose
Mechanisms of data availability: After approval by the EORTC Quality of Life Group
Any additional restrictions: With ethical approval
